# 4-(4-Fluoro­phen­yl)-2-oxo-1,2,5,6-tetra­hydro­benzo[*h*]quinoline-3-carbonitrile

**DOI:** 10.1107/S1600536809017991

**Published:** 2009-05-20

**Authors:** Jinpeng Zhang, Jie Ding, Shu Yan, Liangce Rong, Lichun Xu

**Affiliations:** a Department of Public Health, Xuzhou Medical College, Xuzhou 221000, People’s Republic of China; b College of Chemistry and Chemical Engineering, Xuzhou Normal University, Xuzhou 221116, People’s Republic of China

## Abstract

In the mol­ecule of the title compound, C_20_H_13_FN_2_O, the fluoro­phenyl ring is oriented at a dihedral angle of 72.76 (3)° with respect to the fused benzene ring. In the crystal structure, inter­molecular N—H⋯O, C—H⋯O and C—H⋯F inter­actions link the mol­ecules into chains. π–π contacts between the quinoline and benzene rings [centroid–centroid distance = 3.918 (3) Å] may further stabilize the structure. A weak C—H⋯π inter­action is also present. The O atom and two of the CH_2_ groups of the quinoline ring system are disordered over two positions. The O atom was refined with occupancies of 0.489 (17) and 0.511 (17), while C and H atoms were refined with occupancies of 0.435 (13) and 0.565 (13).

## Related literature

For general background to substituted six-membered lactams, see: Daly (1998 ▶); Plunkett (1994 ▶); Robertson *et al.* (1986 ▶). For bond-length data, see: Allen *et al.* (1987 ▶).
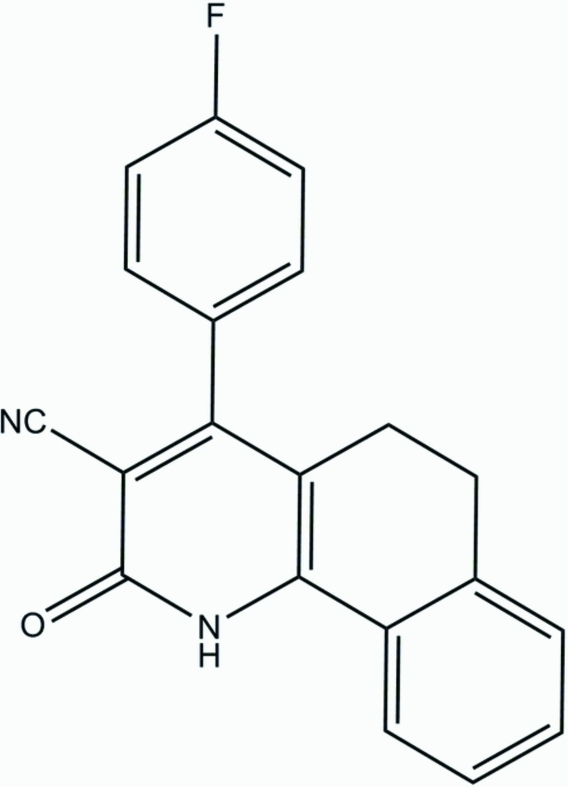


## Experimental

### 

#### Crystal data

C_20_H_13_FN_2_O
*M* *_r_* = 316.32
Triclinic, 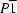

*a* = 8.116 (10) Å
*b* = 9.278 (12) Å
*c* = 11.263 (14) Å
α = 98.674 (19)°
β = 105.095 (17)°
γ = 104.846 (18)°
*V* = 769.7 (16) Å^3^
*Z* = 2
Mo *K*α radiation
μ = 0.09 mm^−1^
*T* = 298 K
0.48 × 0.35 × 0.33 mm

#### Data collection

Bruker SMART CCD area-detector diffractometer
Absorption correction: multi-scan (*SADABS*; Sheldrick, 1996 ▶) *T*_min_ = 0.956, *T*_max_ = 0.970
3950 measured reflections
2656 independent reflections
1399 reflections with *I* > 2σ(*I*)
*R*_int_ = 0.020

#### Refinement

*R*[*F* ^2^ > 2σ(*F* ^2^)] = 0.048
*wR*(*F* ^2^) = 0.144
*S* = 1.00
2656 reflections
240 parameters
H-atom parameters constrained
Δρ_max_ = 0.15 e Å^−3^
Δρ_min_ = −0.17 e Å^−3^

### 

Data collection: *SMART* (Bruker, 1998 ▶); cell refinement: *SAINT* (Bruker, 1998 ▶); data reduction: *SAINT*; program(s) used to solve structure: *SHELXS97* (Sheldrick, 2008 ▶); program(s) used to refine structure: *SHELXL97* (Sheldrick, 2008 ▶); molecular graphics: *ORTEP-3 for Windows* (Farrugia, 1997 ▶); software used to prepare material for publication: *SHELXL97* and *PLATON* (Spek, 2009 ▶).

## Supplementary Material

Crystal structure: contains datablocks global, I. DOI: 10.1107/S1600536809017991/hk2684sup1.cif

Structure factors: contains datablocks I. DOI: 10.1107/S1600536809017991/hk2684Isup2.hkl

Additional supplementary materials:  crystallographic information; 3D view; checkCIF report

## Figures and Tables

**Table 1:** Hydrogen-bond geometry (Å, °)

*D*—H⋯*A*	*D*—H	H⋯*A*	*D*⋯*A*	*D*—H⋯*A*
N1—H1⋯O1^i^	0.86	2.08	2.883 (3)	155
C7—H7⋯O1^i^	0.93	2.35	3.223 (3)	157
C12—H12*B*⋯O1^ii^	0.97	2.21	2.863 (3)	124
C13—H13*B*⋯F1^iii^	0.97	2.42	3.270 (3)	147
C15—H15⋯*Cg*3^iv^	0.93	2.90	3.671 (3)	141

Symmetry codes: (i) 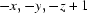; (ii) 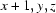; (iii) 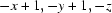; (iv) 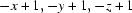.

**Fig. 1.**
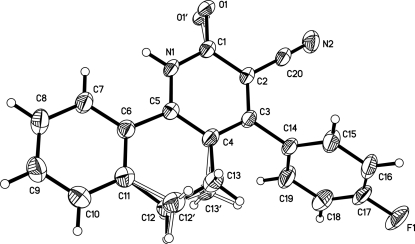
The molecular structure of the title molecule, with the atom-numbering scheme. Displacement ellipsoids are drawn at the 30% probability level.

**Fig. 2.**
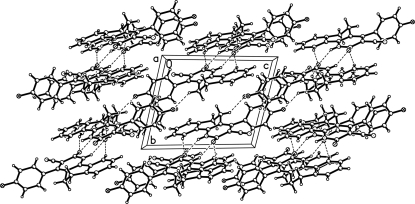
A partial packing diagram of the title compound. Hydrogen bonds are shown as dashed lines.

## supplementary crystallographic information

### Comment

Substituted six-membered lactams have attracted the attention of synthetic organic chemists for many years because these structural features are found in a wide variety of naturally occurring alkaloids (Daly, 1998; Plunkett, 1994). Since compounds with these scaffolds have been shown to exhibit significant pharmacological properties, medicinal chemists often incorporate these motifs in the design of novel biologically active molecules. For example, compounds Arnrinone 1 and Milrinone 2 are the cardiotonic drugs (Robertson *et al.*, 1986) and that have been found to display effective activities on therapy of miocardial infarction. Development of a general and efficient synthetic strategy to synthesize those compounds is still desired. We report herein the crystal structure of the title compound.

In the molecule of the title compound (Fig. 1), the bond lengths (Allen *et al.*,  1987) and angles are within normal ranges. Rings A (N1/C1-C5), C (C6-C11) and D (C14-C19) are, of course, planar and they are oriented at dihedral angles of A/C = 3.57 (3), A/D = 76.11 (3) and C/D = 72.76 (3) °. Ring B (C4-C6/C11-C13) is not planar, and adopts twisted conformation.

In the crystal structure, intermolecular N-H···O, C-H···O and C-H···F interactions (Table 1) link the molecules into chains (Fig. 2), in which they may be effective in the stabilization of the structure. The π···π contact between the quinoline and the benzene rings, Cg1—Cg3^i^ [symmetry code: (i) 1 - x, -y, 1 - z, where Cg1 and Cg3 are centroids of the rings A (N1/C1-C5) and C (C6-C11), respectively] may further stabilize the structure, with centroid-centroid distance of 3.918 (3) Å. There also exists a weak C-H···π interaction (Table 1).

### Experimental

The title compound was prepared by the reaction of 3,4-dihydronaphthalen-1(2H) -one (2 mmol), aromatic aldehydes (2 mmol), malononitrile (3 mmol) and NaOH (2 mmol) under solvent-free conditions using heating method.

### Refinement

The O1, C12, C13, H12A, H12B, H13A and H13B atoms were disordered. During the refinement process, the disordered C and H atoms were refined with occupancies of 0.435 (13) and 0.565 (13), while O atom was refined with occupancies of 0.489 (17) and 0.511 (17). H atoms were positioned geometrically with N-H = 0.86 Å (for NH) and C-H = 0.93 and 0.97 Å, for aromatic and methylene H atoms, respectively, and constrained to ride on their parent atoms, with U_iso_(H) = 1.2U_eq_(C,N).

### Crystal data

**Table:**

C_20_H_13_FN_2_O	*Z* = 2
*M**_r_* = 316.32	*F*(000) = 328
Triclinic, *P*1	*D*_x_ = 1.365 Mg m^−^^3^
Hall symbol: -P 1	Melting point > 598 K
*a* = 8.116 (10) Å	Mo *K*α radiation, λ = 0.71073 Å
*b* = 9.278 (12) Å	Cell parameters from 929 reflections
*c* = 11.263 (14) Å	θ = 2.3–25.4°
α = 98.674 (19)°	µ = 0.09 mm^−^^1^
β = 105.095 (17)°	*T* = 298 K
γ = 104.846 (18)°	Block, colourless
*V* = 769.7 (16) Å^3^	0.48 × 0.35 × 0.33 mm

### Data collection

**Table:**

Bruker SMART CCD area-detector diffractometer	2656 independent reflections
Radiation source: fine-focus sealed tube	1399 reflections with *I* > 2σ(*I*)
graphite	*R*_int_ = 0.020
φ and ω scans	θ_max_ = 25.0°, θ_min_ = 2.3°
Absorption correction: multi-scan (*SADABS*; Sheldrick, 1996)	*h* = −9→9
*T*_min_ = 0.956, *T*_max_ = 0.970	*k* = −11→8
3950 measured reflections	*l* = −9→13

### Refinement

**Table:**

Refinement on *F*^2^	Secondary atom site location: difference Fourier map
Least-squares matrix: full	Hydrogen site location: inferred from neighbouring sites
*R*[*F*^2^ > 2σ(*F*^2^)] = 0.048	H-atom parameters constrained
*wR*(*F*^2^) = 0.144	*w* = 1/[σ^2^(*F*_o_^2^) + (0.0683*P*)^2^] where *P* = (*F*_o_^2^ + 2*F*_c_^2^)/3
*S* = 1.00	(Δ/σ)_max_ < 0.001
2656 reflections	Δρ_max_ = 0.15 e Å^−^^3^
240 parameters	Δρ_min_ = −0.17 e Å^−^^3^
Primary atom site location: structure-invariant direct methods	

### Special details

**Table:**

Geometry. All e.s.d.'s (except the e.s.d. in the dihedral angle between two l.s. planes) are estimated using the full covariance matrix. The cell e.s.d.'s are taken into account individually in the estimation of e.s.d.'s in distances, angles and torsion angles; correlations between e.s.d.'s in cell parameters are only used when they are defined by crystal symmetry. An approximate (isotropic) treatment of cell e.s.d.'s is used for estimating e.s.d.'s involving l.s. planes.
Refinement. Refinement of *F*^2^ against ALL reflections. The weighted *R*-factor *wR* and goodness of fit *S* are based on *F*^2^, conventional *R*-factors *R* are based on *F*, with *F* set to zero for negative *F*^2^. The threshold expression of *F*^2^ > σ(*F*^2^) is used only for calculating *R*-factors(gt) *etc*. and is not relevant to the choice of reflections for refinement. *R*-factors based on *F*^2^ are statistically about twice as large as those based on *F*, and *R*- factors based on ALL data will be even larger.

### Fractional atomic coordinates and isotropic or equivalent isotropic displacement parameters (Å^2^)

**Table:**

	*x*	*y*	*z*	*U*_iso_*/*U*_eq_	Occ. (<1)
F1	0.2899 (2)	0.4261 (2)	−0.18711 (16)	0.1127 (7)	
N1	0.2029 (3)	0.1315 (2)	0.45914 (18)	0.0580 (6)	
H1	0.1926	0.0948	0.5235	0.070*	
N2	−0.2136 (3)	0.1660 (3)	0.0916 (2)	0.0781 (7)	
O1	−0.101 (3)	0.0761 (19)	0.3875 (16)	0.063 (2)	0.489 (17)
O1'	−0.100 (2)	0.0203 (19)	0.3604 (16)	0.063 (2)	0.511 (17)
C1	0.0482 (3)	0.1170 (3)	0.3667 (2)	0.0589 (7)	
C2	0.0742 (3)	0.1847 (3)	0.2646 (2)	0.0474 (6)	
C3	0.2423 (3)	0.2493 (3)	0.2576 (2)	0.0449 (6)	
C4	0.3947 (3)	0.2594 (3)	0.3564 (2)	0.0514 (7)	
C5	0.3716 (3)	0.1991 (3)	0.4576 (2)	0.0441 (6)	
C6	0.5250 (3)	0.2014 (3)	0.5626 (2)	0.0472 (6)	
C7	0.5053 (4)	0.1393 (3)	0.6645 (2)	0.0611 (7)	
H7	0.3908	0.0975	0.6694	0.073*	
C8	0.6514 (4)	0.1387 (3)	0.7579 (2)	0.0673 (8)	
H8	0.6354	0.0973	0.8259	0.081*	
C9	0.8191 (4)	0.1978 (3)	0.7521 (3)	0.0705 (8)	
H9	0.9181	0.1954	0.8149	0.085*	
C10	0.8420 (4)	0.2613 (4)	0.6529 (3)	0.0817 (9)	
H10	0.9575	0.3021	0.6496	0.098*	
C11	0.6977 (4)	0.2660 (3)	0.5580 (2)	0.0652 (8)	
C12	0.7151 (13)	0.2943 (16)	0.4310 (10)	0.060 (2)	0.435 (13)
H12A	0.8345	0.3611	0.4429	0.072*	0.435 (13)
H12B	0.6964	0.1979	0.3740	0.072*	0.435 (13)
C13	0.5770 (13)	0.3675 (14)	0.3755 (13)	0.061 (3)	0.435 (13)
H13A	0.5955	0.4640	0.4323	0.073*	0.435 (13)
H13B	0.5872	0.3880	0.2953	0.073*	0.435 (13)
C12'	0.7237 (10)	0.3759 (13)	0.4687 (7)	0.069 (2)	0.565 (13)
H12C	0.7097	0.4729	0.5027	0.083*	0.565 (13)
H12D	0.8432	0.3954	0.4608	0.083*	0.565 (13)
C13'	0.5852 (10)	0.3014 (12)	0.3417 (7)	0.059 (2)	0.565 (13)
H13C	0.6068	0.2095	0.3046	0.071*	0.565 (13)
H13D	0.5936	0.3710	0.2858	0.071*	0.565 (13)
C14	0.2601 (3)	0.3026 (3)	0.1417 (2)	0.0493 (6)	
C15	0.2276 (4)	0.4341 (3)	0.1176 (3)	0.0681 (8)	
H15	0.1976	0.4956	0.1764	0.082*	
C16	0.2388 (4)	0.4774 (4)	0.0065 (3)	0.0769 (9)	
H16	0.2165	0.5671	−0.0098	0.092*	
C17	0.2827 (4)	0.3865 (4)	−0.0771 (3)	0.0729 (9)	
C18	0.3162 (4)	0.2565 (4)	−0.0566 (3)	0.0858 (10)	
H18	0.3456	0.1957	−0.1163	0.103*	
C19	0.3064 (4)	0.2146 (4)	0.0539 (3)	0.0769 (9)	
H19	0.3314	0.1256	0.0696	0.092*	
C20	−0.0853 (4)	0.1739 (3)	0.1674 (2)	0.0561 (7)	

### Atomic displacement parameters (Å^2^)

**Table:**

	*U*^11^	*U*^22^	*U*^33^	*U*^12^	*U*^13^	*U*^23^
F1	0.0932 (13)	0.1675 (19)	0.0723 (12)	0.0028 (12)	0.0250 (10)	0.0801 (12)
N1	0.0489 (13)	0.0912 (17)	0.0447 (12)	0.0206 (12)	0.0199 (11)	0.0405 (12)
N2	0.0698 (17)	0.100 (2)	0.0663 (16)	0.0261 (15)	0.0106 (14)	0.0435 (15)
O1	0.0480 (12)	0.100 (8)	0.058 (6)	0.025 (5)	0.027 (3)	0.044 (5)
O1'	0.0480 (12)	0.100 (8)	0.058 (6)	0.025 (5)	0.027 (3)	0.044 (5)
C1	0.0495 (16)	0.088 (2)	0.0505 (16)	0.0231 (15)	0.0207 (14)	0.0367 (15)
C2	0.0511 (15)	0.0574 (16)	0.0420 (14)	0.0187 (13)	0.0187 (12)	0.0256 (12)
C3	0.0535 (15)	0.0467 (15)	0.0418 (14)	0.0153 (12)	0.0212 (12)	0.0207 (12)
C4	0.0509 (15)	0.0569 (16)	0.0503 (15)	0.0113 (13)	0.0198 (13)	0.0266 (13)
C5	0.0461 (14)	0.0482 (15)	0.0414 (14)	0.0141 (12)	0.0164 (12)	0.0163 (12)
C6	0.0485 (15)	0.0505 (16)	0.0426 (14)	0.0140 (13)	0.0143 (12)	0.0131 (12)
C7	0.0549 (17)	0.078 (2)	0.0479 (16)	0.0119 (14)	0.0124 (13)	0.0282 (14)
C8	0.072 (2)	0.068 (2)	0.0521 (17)	0.0144 (16)	0.0056 (16)	0.0245 (15)
C9	0.064 (2)	0.080 (2)	0.0585 (19)	0.0225 (17)	0.0024 (15)	0.0179 (16)
C10	0.0517 (18)	0.114 (3)	0.080 (2)	0.0235 (18)	0.0158 (16)	0.037 (2)
C11	0.0536 (17)	0.085 (2)	0.0588 (17)	0.0193 (15)	0.0158 (14)	0.0276 (16)
C12	0.043 (4)	0.072 (6)	0.068 (5)	0.015 (5)	0.020 (4)	0.026 (4)
C13	0.065 (5)	0.062 (6)	0.057 (5)	0.012 (4)	0.024 (4)	0.024 (4)
C12'	0.055 (3)	0.079 (5)	0.071 (4)	0.010 (4)	0.021 (3)	0.029 (4)
C13'	0.049 (3)	0.071 (5)	0.062 (5)	0.010 (4)	0.024 (3)	0.032 (4)
C14	0.0499 (15)	0.0571 (16)	0.0440 (15)	0.0110 (13)	0.0178 (12)	0.0253 (13)
C15	0.084 (2)	0.0655 (19)	0.0653 (19)	0.0249 (16)	0.0280 (16)	0.0360 (15)
C16	0.077 (2)	0.075 (2)	0.079 (2)	0.0134 (17)	0.0159 (18)	0.0533 (18)
C17	0.0537 (18)	0.108 (3)	0.0502 (18)	−0.0009 (18)	0.0128 (14)	0.0486 (19)
C18	0.104 (3)	0.112 (3)	0.064 (2)	0.038 (2)	0.0474 (19)	0.041 (2)
C19	0.112 (3)	0.086 (2)	0.0628 (19)	0.046 (2)	0.0480 (19)	0.0436 (17)
C20	0.0602 (18)	0.0676 (18)	0.0495 (17)	0.0195 (15)	0.0222 (15)	0.0318 (14)

### Geometric parameters (Å, °)

**Table:**

F1—C17	1.356 (3)	C10—C11	1.381 (4)
N1—C5	1.358 (3)	C10—H10	0.9300
N1—C1	1.367 (3)	C11—C12	1.528 (9)
N1—H1	0.8600	C11—C12'	1.550 (8)
N2—C20	1.139 (3)	C12—C13	1.505 (14)
O1—C1	1.267 (18)	C12—H12A	0.9700
O1'—C1	1.279 (18)	C12—H12B	0.9700
C1—C2	1.428 (3)	C13—H13A	0.9700
C2—C3	1.370 (3)	C13—H13B	0.9700
C2—C20	1.431 (4)	C12'—C13'	1.501 (12)
C3—C4	1.404 (3)	C12'—H12C	0.9700
C3—C14	1.491 (3)	C12'—H12D	0.9700
C4—C5	1.377 (3)	C13'—H13C	0.9700
C4—C13	1.499 (10)	C13'—H13D	0.9700
C4—C13'	1.553 (8)	C14—C15	1.363 (4)
C5—C6	1.470 (3)	C14—C19	1.376 (4)
C6—C7	1.387 (3)	C15—C16	1.389 (4)
C6—C11	1.394 (4)	C15—H15	0.9300
C7—C8	1.367 (4)	C16—C17	1.347 (4)
C7—H7	0.9300	C16—H16	0.9300
C8—C9	1.354 (4)	C17—C18	1.342 (4)
C8—H8	0.9300	C18—C19	1.375 (4)
C9—C10	1.371 (4)	C18—H18	0.9300
C9—H9	0.9300	C19—H19	0.9300
			
C5—N1—C1	125.3 (2)	C13—C12—H12A	109.9
C5—N1—H1	117.4	C11—C12—H12A	109.9
C1—N1—H1	117.4	C13—C12—H12B	109.9
O1—C1—N1	119.7 (9)	C11—C12—H12B	109.9
O1'—C1—N1	120.2 (8)	H12A—C12—H12B	108.3
O1—C1—C2	124.0 (9)	C4—C13—C12	108.2 (9)
O1'—C1—C2	123.2 (8)	C4—C13—H13A	110.1
N1—C1—C2	114.7 (2)	C12—C13—H13A	110.1
C3—C2—C1	121.7 (2)	C4—C13—H13B	110.1
C3—C2—C20	122.2 (2)	C12—C13—H13B	110.1
C1—C2—C20	116.0 (2)	H13A—C13—H13B	108.4
C2—C3—C4	120.1 (2)	C13'—C12'—C11	108.2 (7)
C2—C3—C14	119.1 (2)	C13'—C12'—H12C	110.1
C4—C3—C14	120.8 (2)	C11—C12'—H12C	110.1
C5—C4—C3	118.8 (2)	C13'—C12'—H12D	110.1
C5—C4—C13	116.3 (5)	C11—C12'—H12D	110.1
C3—C4—C13	122.8 (5)	H12C—C12'—H12D	108.4
C5—C4—C13'	118.0 (4)	C12'—C13'—C4	109.8 (7)
C3—C4—C13'	121.8 (4)	C12'—C13'—H13C	109.7
N1—C5—C4	119.4 (2)	C4—C13'—H13C	109.7
N1—C5—C6	118.8 (2)	C12'—C13'—H13D	109.7
C4—C5—C6	121.8 (2)	C4—C13'—H13D	109.7
C7—C6—C11	118.7 (2)	H13C—C13'—H13D	108.2
C7—C6—C5	122.9 (2)	C15—C14—C19	118.4 (2)
C11—C6—C5	118.4 (2)	C15—C14—C3	122.3 (2)
C8—C7—C6	121.1 (3)	C19—C14—C3	119.2 (2)
C8—C7—H7	119.5	C14—C15—C16	120.9 (3)
C6—C7—H7	119.5	C14—C15—H15	119.5
C9—C8—C7	120.4 (3)	C16—C15—H15	119.5
C9—C8—H8	119.8	C17—C16—C15	118.4 (3)
C7—C8—H8	119.8	C17—C16—H16	120.8
C8—C9—C10	119.5 (3)	C15—C16—H16	120.8
C8—C9—H9	120.2	C18—C17—C16	122.5 (3)
C10—C9—H9	120.2	C18—C17—F1	118.7 (3)
C9—C10—C11	121.6 (3)	C16—C17—F1	118.8 (3)
C9—C10—H10	119.2	C17—C18—C19	118.9 (3)
C11—C10—H10	119.2	C17—C18—H18	120.5
C10—C11—C6	118.7 (3)	C19—C18—H18	120.5
C10—C11—C12	121.7 (4)	C18—C19—C14	120.9 (3)
C6—C11—C12	117.1 (4)	C18—C19—H19	119.6
C10—C11—C12'	120.9 (4)	C14—C19—H19	119.6
C6—C11—C12'	118.5 (4)	N2—C20—C2	178.8 (3)
C13—C12—C11	108.8 (9)		

### Hydrogen-bond geometry (Å, °)

**Table:**

*D*—H···*A*	*D*—H	H···*A*	*D*···*A*	*D*—H···*A*
N1—H1···O1^i^	0.86	2.08	2.883 (3)	155
C7—H7···O1^i^	0.93	2.35	3.223 (3)	157
C12—H12B···O1^ii^	0.97	2.21	2.863 (3)	124
C13—H13B···F1^iii^	0.97	2.42	3.270 (3)	147
C15—H15···Cg3^iv^	0.93	2.90	3.671 (3)	141

Symmetry codes: (i) −*x*, −*y*, −*z*+1; (ii) *x*+1, *y*, *z*; (iii) −*x*+1, −*y*+1, −*z*; (iv) −*x*+1, −*y*+1, −*z*+1.
